# Atrial fibrillation accelerates functional decline in older adults: a 15-year follow-up population-based study

**DOI:** 10.1093/europace/euae173

**Published:** 2024-06-24

**Authors:** Chukwuma Okoye, Chengxuan Qiu, Xin Xia, Gregory Yoke Hong Lip, Giuseppe Bellelli, Anna-Karin Welmer, Amaia Calderón-Larrañaga, Davide Liborio Vetrano

**Affiliations:** Aging Research Center, Department of Neurobiology, Care Sciences and Society (NVS), Karolinska Institutet, Stockholm University, Tomtebodavägen 18a, 171 65 Solna, Sweden; Department of Medicine and Surgery, University of Milan-Bicocca, Via Cadore 48, 20900 Monza Italy; Acute Geriatric Unit, IRCCS Foundation San Gerardo dei Tintori, Monza, Via Pergolesi 33, 20900 Monza, Italy; Aging Research Center, Department of Neurobiology, Care Sciences and Society (NVS), Karolinska Institutet, Stockholm University, Tomtebodavägen 18a, 171 65 Solna, Sweden; Division of Neurogeriatrics, Department of NVS, Karolinska Institutet, Stockholm, Sweden; Liverpool Centre for Cardiovascular Science, University of Liverpool, Liverpool, UK; Liverpool John Moores University, Liverpool, UK; Liverpool Heart and Chest Hospital, Liverpool, UK; Danish Center for Health Services Research, Department of Clinical Medicine, Aalborg University, Aalborg, Denmark; Department of Medicine and Surgery, University of Milan-Bicocca, Via Cadore 48, 20900 Monza Italy; Acute Geriatric Unit, IRCCS Foundation San Gerardo dei Tintori, Monza, Via Pergolesi 33, 20900 Monza, Italy; Aging Research Center, Department of Neurobiology, Care Sciences and Society (NVS), Karolinska Institutet, Stockholm University, Tomtebodavägen 18a, 171 65 Solna, Sweden; Division of Physiotherapy, Department of Neurobiology, Care Sciences and Society, Karolinska Institutet, Stockholm, Sweden; Stockholm Gerontology Research Centre, Stockholm, Sweden; Women’s Health and Allied Health Professionals Theme, Medical Unit Medical Psychology, Karolinska University Hospital, Stockholm, Sweden; Aging Research Center, Department of Neurobiology, Care Sciences and Society (NVS), Karolinska Institutet, Stockholm University, Tomtebodavägen 18a, 171 65 Solna, Sweden; Aging Research Center, Department of Neurobiology, Care Sciences and Society (NVS), Karolinska Institutet, Stockholm University, Tomtebodavägen 18a, 171 65 Solna, Sweden

**Keywords:** Atrial fibrillation, Older persons, Physical performance, Walking speed, Oral anticoagulation therapy

## Abstract

**Aims:**

Atrial fibrillation (AF) has been associated with functional impairment. However, the role exerted by AF on the long-term trajectories of functional mobility remains to be elucidated. This study aimed to evaluate the impact of AF on functional mobility by tracing walking speed (WS) trajectories over 15 years of follow-up in a population-based cohort of individuals aged 60+ years.

**Methods and results:**

This population-based cohort study included 3141 community-dwelling participants (mean age 73.7 years; 63.6% women) from the Swedish National Study on Aging and Care in Kungsholmen, who were regularly examined from 2001–2004 to 2016–2019. Functional mobility was assessed by measuring WS in a standardized way. The association between AF and WS trajectories was assessed by multivariable joint models accounting for the longitudinal dropouts due to death. Stratified analyses by demographic and clinical factors were performed. The effect-modifying role of oral anticoagulant therapy (OAC), incident heart failure (HF), and incident stroke was finally investigated. At baseline, 285 (9.1%) participants were ascertained to have AF. A faster annual WS decline was observed in persons with AF than in non-AF peers (adjusted *β* coefficient per year = −0.011, 95% confidence interval: −0.016 to −0.005). Incident HF and stroke were associated with greater WS decline in participants with AF. OAC use was not associated with a slower functional decline.

**Conclusion:**

Atrial fibrillation is associated with a faster physical function decline in older individuals. Incident HF and stroke possibly accelerate WS decline over time in AF participants.

## Graphical Abstract

**Graphical Abstract:**
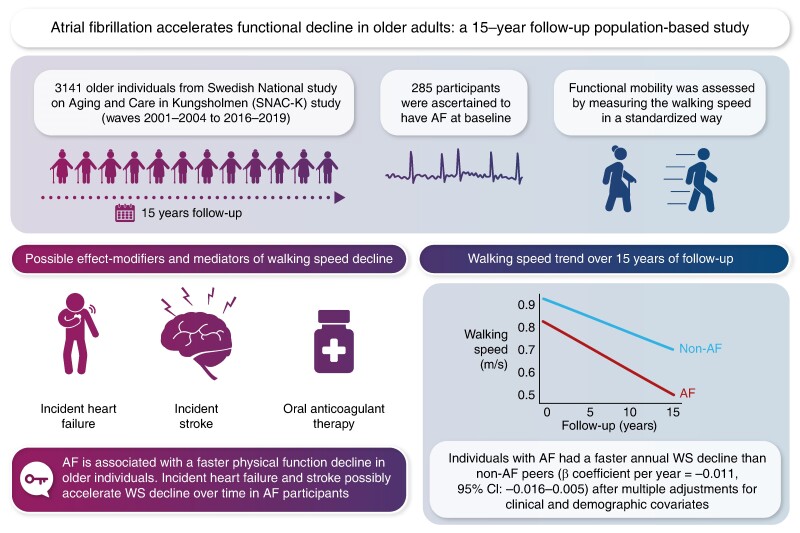

**What’s new?**

**Key findings**

- Over a 15-year follow-up study, older adults living in the community with atrial fibrillation exhibited a more rapid annual decline in walking speed compared with those without atrial fibrillation.
- Among individuals with atrial fibrillation, a faster decline in walking speed was notably linked with incident stroke and incident heart failure.
- The use of oral anticoagulant therapy did not demonstrate an association with a slower decline in walking speed.

**Take-home message**

Atrial fibrillation is associated with an accelerated physical function decline in older individuals. Incident heart failure and stroke may further hasten the decline in walking speed decline over time among those with atrial fibrillation.

## Background

Atrial fibrillation (AF) is the most common arrhythmia worldwide, and its prevalence increases progressively with age.^1^ Due to the extended human longevity, it has been estimated that one out of the three persons of European ancestry at index age of 55 years would develop AF.^2^ Individuals with AF often display characteristics of frailty, carry a higher burden of comorbidity, and exhibit a greater susceptibility to poor health outcomes when compared with those without AF.^1–4^ While extensive research has been conducted concerning the impact of AF on cardiovascular events, emerging interest lies in understanding its potential associations with non-cardiovascular outcomes, such as functional mobility.^5^ Functional mobility is the physiological ability of people to move independently and safely in a variety of environments to accomplish functional activities or tasks and to participate in activities of daily living (ADL), at home, at work, and in the community.^6^ The possible relationship between AF and functional mobility involves numerous interconnected pathways.^7^ Specifically, AF has been associated with reduced cerebral blood flow, increased stroke risk, and a higher burden of comorbidities, potentially resulting in cognitive impairment and deficits in motor control.^7–9^ Additionally, AF is linked with frailty, and the decreased physiological reserve of this condition may increase the energy cost while walking, further reducing functional mobility.^8^ Finally, individuals with AF are more often on polypharmacotherapy and present with low levels of physical activity, all factors that can negatively influence functional mobility.^10,11^ Understanding the associations between those factors and physical performance in older adults with AF can contribute to early detection, prevention, and tailored interventions aimed at minimizing physical function decline in older adults with AF.

The walking speed (WS) test has been widely recognized as a good surrogate of physical function.^12^ It combines complex mechanisms of balance and energy, demanding the correct functioning of multiple organs and the musculoskeletal system.^7,9^ Deficits in WS are associated with adverse outcomes, such as hospitalizations, increased risk of falls, cognitive decline, and death.^7,9,10,12,13^ To date, however, few investigations have characterized WS trajectories associated with AF in older individuals^8,14^; none has sought to determine the effect of AF-related incident events on WS decline over time.

In this study, we sought to evaluate the association of AF with physical function decline using data from a longitudinal study of community-dwelling older adults who have been followed up for 15 years.^15^ Our specific objectives were (i) to evaluate the association of prevalent AF with the motor function decline over 15 years; (ii) to determine the interaction between AF-related incident events, including heart failure (HF), stroke, and dementia, and WS decline over time in older individuals with AF; and (iii) to determine the possible effect of oral anticoagulant therapy (OAC) on the WS decline among participants with AF.

## Methods

### Study population

Data were collected from the Swedish National Study on Aging and Care in Kungsholmen (SNAC-K), which is an ongoing population-based study of community-dwelling and institutionalized older adults ≥60 years. The SNAC-K study involved 11 age cohorts at baseline, and they were assigned to younger old groups (60, 66, and 72 years) and older old groups (78, 81, 84, 87, 90, 93, 96, and ≥99 years) living in the Kungsholmen district (Stockholm, Sweden). Those who accepted the invitation were evaluated for the first time between 2001 and 2004 and then followed up every 6 years for those aged <78 years or every 3 years for those aged ≥78 years. At each study wave, SNAC-K participants undergo an approximately 5-hour comprehensive clinical and functional assessment carried out by trained physicians, nurses, and neuropsychologists. Physicians collect information on diagnoses of disorders or health conditions via physical examinations, medical history, examination of medical charts, self-reported information, and/or proxy interviews.

For the current study, we utilized 15-year follow-up data from 2001–2004 (Wave 1) to 2016–2019 (Wave 6). At baseline, 3363 people were examined (participation rate, 73%). Of these, we excluded individuals institutionalized at baseline (*n* = 191) and participants with no follow-up WS measurement (*n* = 31), leaving 3141 participants for the current analysis. Supplementary material online, *Figure S1*, shows the flowchart of the study participants.

All parts of the SNAC-K study (including linkage with the patient and death registers) were approved by the regional ethical review board in Stockholm. Written informed consent was obtained from all participants or, in case of persons with cognitive impairment, from proxies (next of kin or guardians).

### Ascertainment of atrial fibrillation

Atrial fibrillation was diagnosed at baseline through a physician’s examination and electrocardiogram (ECG), where discrete P waves were undetectable and irregular ventricular rate was observed on a 12-lead ECG.^15^ In addition, the Swedish National Patient Register, which includes comprehensive records from hospital and specialist outpatient care, was reviewed to identify the presence and onset date of AF in patients with a known history of the condition.

### Walking speed assessment

To assess WS, participants were asked to walk 6 m at their usual speed at each study visit, or alternatively, 2.4 m when participants reported that they walked slowly or when the assessment was carried out in restricted spaces. Walking speed was reported in metre per second.

### Assessment of covariates

Educational attainment was ascertained by nurses through interviews and categorized as elementary, high school, and university or higher. Body mass index (BMI) was obtained by dividing the participants’ weight in kilograms by their squared height in metres. Physical activity was divided into inadequate (light exercise ≤2–3 times per month), health enhancing (moderate exercise ≤2–3 times per month), and fitness enhancing (intensive exercise several times per week). Smoking habits were obtained by nurse interview and categorized as current/former smoker or never smoked. Alcohol consumption was categorized as no/occasional, light-to-moderate (1–14 drinks per week for men or 1–7 drinks per week for women), or heavy (>14 drinks per week for men or >7 drinks per week for women) drinking. The assessment of basic activities of daily living (B-ADL)^16^ was conducted through nurse interviews, evaluating impairment across six domains: bathing, dressing, toileting, continence, transferring from bed, and eating. Impairment levels were graded on a scale from 0 (indicating independence in all ADLs) to 6 (indicating the need for support in all six ADL domains). Similarly, instrumental activities of daily living (I-ADL)^17^were assessed on a scale ranging from 0 (reflecting independence in all IADL) to 8 (indicating the need for support in all eight IADL domains). These encompass a person’s capability in food preparation, medication management, shopping, communication, financial management, housekeeping, transportation, and laundry. Global cognitive function was measured with the Mini-Mental State Examination (MMSE).^18^

The clinical ascertainment and operationalization of chronic diseases in SNAC-K are reported elsewhere.^19^ The chronic use of OAC was assessed selecting patients who were receiving warfarin (ATC code BA01AA03) as home therapy for AF. Incident stroke was defined as first-ever stroke occurring over the follow-up period among the stroke-free participants at baseline. The occurrence of stroke was ascertained through linkage to the Swedish National Patient Register.^19^ Dementia was diagnosed at each wave according to the Diagnostic and Statistical Manual of Mental Disorders (4th edition) criteria, using a validated three-step procedure.^20^ Incident HF was assessed at each wave by the SNAC-K physician based on clinical interviews and review of data from the Swedish National Patient Register.^19^

### Statistical analysis

The characteristics of the study population were summarized and reported as means and SD, or medians and interquartile ranges (IQR) for non-normally distributed continuous variables, and as frequencies and percentages for categorical variables.

To evaluate the effect of AF on WS decline across the 15-year follow-up period, we ran multivariable joint models accounting for non-random attrition due to death.^21^ Such joint models consist of two sub-models which were fitted simultaneously: a linear mixed-effects model with WS as the outcome and a Cox proportional hazard model with death as the outcome of interest. In addition, through the calculation of the association parameter α, joint modelling enables the estimation of the effect of the predictor (WS) on the hazard of mortality. In the linear mixed-effects sub-model, fixed effects included AF status, time, and the interaction between the two (AF × time). Random effects included a random intercept to allow for individual differences at baseline.

The linear mixed-effects models were first adjusted for sex, age, and education (Model 1) and then further for hypertension, stroke, HF, chronic obstructive pulmonary disease, dementia, diabetes, physical activity, alcohol consumption, and BMI (Model 2). We also performed three-level interaction analyses and stratified analyses by demographic factors (sex and age), lifestyle factors (physical activity levels), and comorbidities (HF, history of stroke, chronic obstructive pulmonary disease, diabetes, and hypertension) to evaluate whether the effects of AF on WS decline could be modified by these factors or health conditions.

To assess the robustness of the associations between AF and WS decline, we conducted two sensitivity analyses. Firstly, to examine participants without significant frailty or impaired functional status at baseline, we performed three-level interaction analyses on a subset of individuals with a baseline WS greater than 0.5 m/s. Secondly, to mitigate potential confounding in AF participants, propensity scores were computed for each participant and incorporated into the joint model. These propensity scores included age, sex, education attainment, I-ADL, levels of physical activity, history of HF, type 2 diabetes mellitus, ischaemic heart disease, alcohol consumption, baseline WS, chronic obstructive pulmonary disease, BMI, chronic kidney disease (CKD), diagnosis of dementia at baseline, and history of stroke. The variables were selected upon clinical relevance and statistical significance in *Table 1*.

**Table 1:** Characteristics of the study population

Characteristics	All participants (*n* = 3141)	Non-AF (*n* = 2856)	AF (*n* = 285)	*P* value
Age, mean (SD)	73.7 (10.7)	73.0 (10.5)	81.2 (9.5)	0.015
Female sex, ***n*** (%)	2000 (63.6)	1837 (64.3)	163 (57.2)	<0.001
Education, ***n*** (%)				<0.001
Elementary	518 (16.4)	457 (16.0)	61 (21.4)	
High school	1551 (49.4)	1397 (48.9)	154 (54.0)	
University	1060 (33.7)	995 (34.8)	65 (22.8)	
Smoking habits, ***n*** (%)				0.129
Never	1454 (46.3)	1321 (46.2)	133 (46.6)	
Former/current	1655 (52.7)	1509 (52.8)	146 (51.2)	
Alcohol consumption, ***n*** (%)				0.005
Never/occasionally	1096 (35.2)	974 (34.4)	122 (44.3)	
Light/moderate	1507 (48.5)	1381 (48.7)	126 (45.8)	
Heavy	503 (16.2)	476 (16.8)	27 (9.8)	
Physical activity, ***n*** (%)				<0.001
Inadequate	963 (30.6)	835 (29.2)	128 (44.9)	
Health enhancing	1517 (48.2)	1395 (48.8)	122 (42.8)	
Fitness enhancing	661 (21.0)	626 (21.9)	35 (12.3)	
BMI, ***n*** (%)				0.001
BMI 18.5–25	1311 (41.7)	1198 (41.9)	113 (39.6)	
BMI > 25	1569 (49.9)	1440 (50.4)	129 (45.2)	
BMI < 18.5	80 (2.5)	64 (2.2)	16 (5.6)	
MMSE score, median (IQR)	29 (2)	29 (2)	28 (3)	<0.001
1 + impaired B-ADL (%)	39 (1.2)	32 (1.1)	7 (2.5)	0.09
1 + impaired I-ADL (%)	270 (8.5)	217 (8.3)	53 (19.3)	<0.001
# comorbidities, median (IQR)	4 (3)	3 (3)	5 (3)	<0.001
Hypertension, ***n*** (%)	2192 (69.8)	1992 (69.7)	200 (70.2)	0.778
Chronic obstructive pulmonary disease, ***n*** (%)	151 (4.8)	127 (4.4)	24 (8.4)	0.003
Stroke, ***n*** (%)	214 (6.8)	158 (5.5)	56 (19.6)	<0.001
Diabetes mellitus, ***n*** (%)	278 (8.8)	240 (8.4)	38 (13.3)	<0.001
HF, ***n*** (%)	297 (9.4)	177 (6.2)	120 (42.0)	<0.001
Ischaemic heart disease, ***n*** (%)	460 (14.6)	376 (13.1)	84 (29.5)	<0.001
CKD, ***n*** (%)	1059 (33.7)	905 (31.6)	154 (54.0)	<0.001
Dementia, ***n*** (%)	157 (4.9)	127 (4.4)	30 (10.5)	<0.001
WS, mean (SD)	0.98 (0.45)	1.02 (0.44)	0.71 (0.43)	<0.001
WS < 0.8 ms/s, ***n*** (%)	850 (27.3)	703 (24.8)	147 (51.9)	<0.001
Death, ***n*** (%)	1752 (55.7)	1497 (47.6)	255 (89.5)	<0.001

AF: atrial fibrillation; B-ADL, basic activities of daily living; BMI: body mass index; CKD, chronic kidney disease; HF, heart failure; I-ADL, instrumental activities of daily; IQR, interquartile range; MMSE: Mini-Mental State Examination; SD, standard deviation; WS: walking speed.

Additionally, in a subsample of individuals with AF free from dementia, history of stroke, and HF, we ran a univariable and multivariable logistic regression (using age, sex, education, HF, chronic obstructive pulmonary disease, diabetes, hypertension, and physical activity levels as covariates) to examine the association between AF (exposure) and AF-related incidents event (i.e. incident HF, incident stroke, and incident dementia). Secondly, to estimate the effect of those AF-related incident events on WS decline, we further performed three joint models using incident HF and incident stroke (calculated as time-varying variables) and their interaction with time as fixed effects, based on the same adjustment strategy as indicated for Models 1 and 2.

To evaluate the effect of OAC on WS decline, we performed one last joint model with OAC, time, and their interaction as fixed effects and the intercept as random effects using the aforementioned Models 1 and 2 for the multivariable adjustment. Oral anticoagulant therapy use was categorized into three subgroups: OAC users, non-OAC users, and non-AF participants as controls.

Finally, an alluvial plot was constructed to visually depict transitions over the 15-year follow-up period among participants with AF and those without AF. This plot illustrates changes in states based on normal WS (≥0.8 m/s) and slow WS (<0.8 m/s), along with occurrences of death and dropouts at each follow-up.

The level of significance was defined as two-tailed *P* value < 0.05. All analyses were performed using R software, version 4.1.0, packages: tidyr, mgcv, lme4, JM, survival, ggplot, ggalluvial, and car (RStudio, Inc., Boston, MA, USA).

## Results

**Figure 1:**
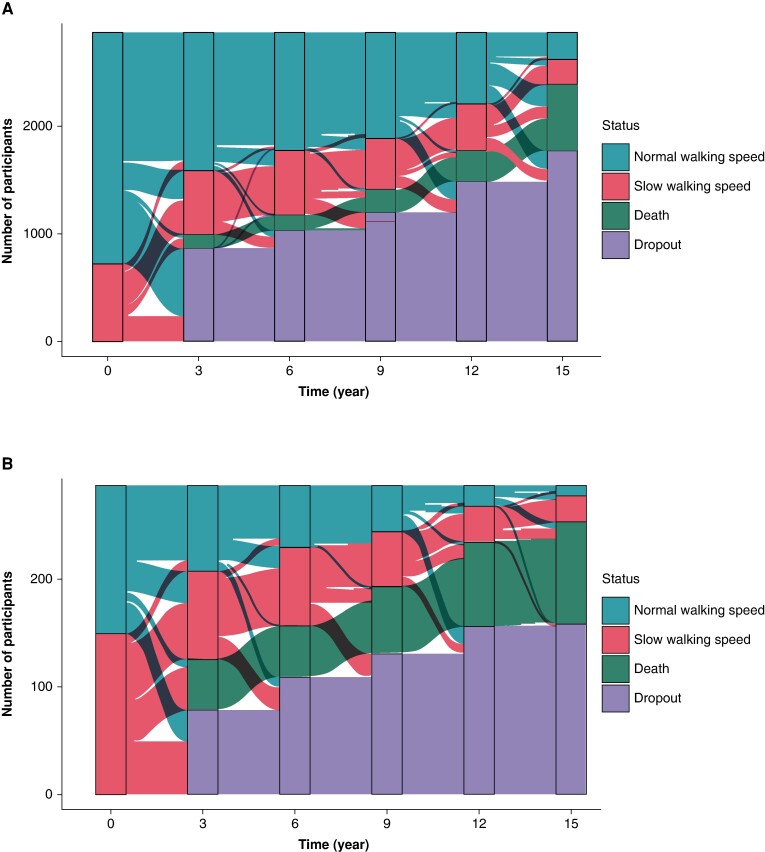
Transitions of WS measurements over 15 years of follow-up among individuals without AF (*A*) and with AF (*B*). AF: atrial fibrillation; WS: walking speed.

Baseline characteristics of the study population are shown in *Table 1*. Of the 3141 participants, 285 (9.1%) had a diagnosis of AF at baseline. Compared with non-AF participants, those with AF were older, with lower levels of education, and a higher burden of comorbidities. Moreover, older adults with AF were more likely to have a history of stroke, diabetes, HF, and dementia at baseline. As depicted in *Figure 1*, participants with AF exhibited a propensity towards slower baseline WS compared with those without AF.

Participants with AF had a faster WS decline than non-AF peers [*β* coefficient per year = −0.011, 95% confidence interval (CI): −0.016 to −0.005] (*Table 2*), a trend consistent with the results obtained in the propensity score–adjusted joint model (see Supplementary material online, *Table S1*). Furthermore, by joint modelling, we confirmed that a decrease of WS over time was significantly associated with an increased mortality risk, irrespective of the AF status (see Supplementary material online, *Table S2*).

**Table 2:** Association between AF and WS accounting for the competing risk of death (joint models)

	Intercept	Annual change in WS
	*β* (95% CI)	*β* (95% CI)
Crude	−0.32 (−0.27; −0.22)	−0.010 (−0.015, −0.004)
Model 1	−0.11 (−0.16; −0.04)	−0.010 (−0.015, −0.004)
Model 2	−0.02 (−0.06; 0.02)	−0.011 (−0.016, −0.005)

Model 1: age + education + sex.

Model 2: age + education + sex + hypertension + chronic obstructive pulmonary disease + HF + stroke + dementia + BMI + diabetes + physical activity + alcohol consumption.

AF: atrial fibrillation; BMI, body mass index; CI: confidence interval; HF, heart failure; WS, walking speed.

**Figure 2:**
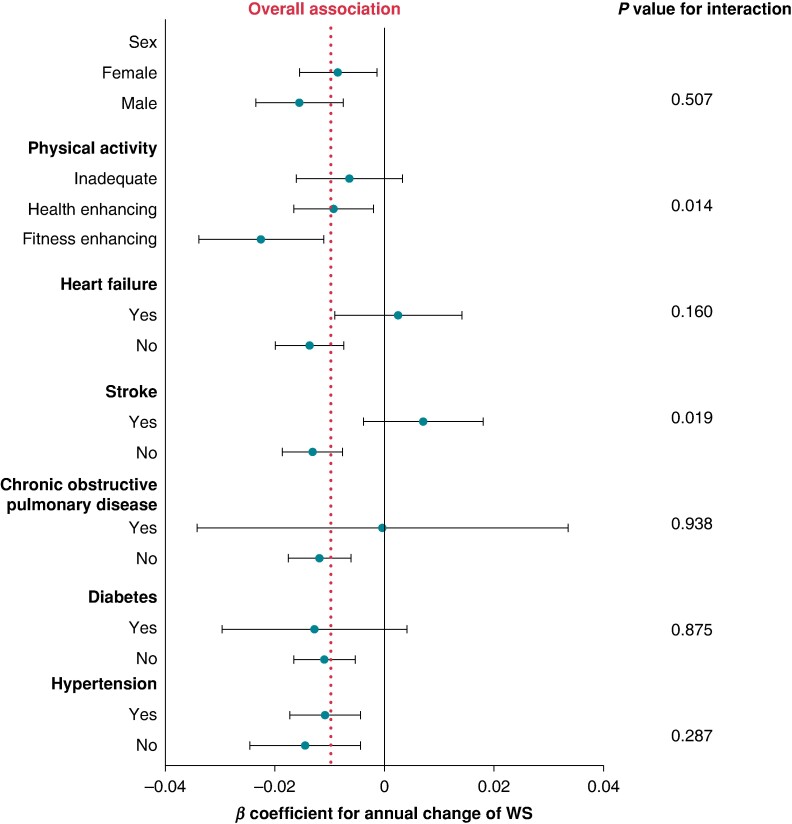
Association between AF and WS accounting for the competing risk of death (Model 2, joint models), stratified by sex, age, heart failure, stroke, chronic obstructive pulmonary disease, diabetes, levels of physical activity, and hypertension. AF: atrial fibrillation; WS: walking speed.

The three-level interaction analyses showed that the impact of AF on WS decline was more pronounced in persons without a history of stroke (*P*_interaction_ = 0.019) and in those with a higher baseline-reported physical activity (*P*_interaction_ = 0.014) (*Figure 2*). In a subsample of participants with WS faster than 0.5 m/s, those conditions were not confirmed as significantly associated with a higher WS decline (*P*_interaction_ > 0.05) (see Supplementary material online, *Tables S3* and *S4*).

During the 15-year follow-up, older adults with AF had a higher incidence of stroke (17.6 vs. 9.4%) and HF (25 vs. 11.5%), compared with their peers. In multivariate logistic analysis, AF was associated with increased odds of incident HF and incident stroke; on the contrary, the relationship between AF and incident dementia was not confirmed after adjustment (see Supplementary material online, *Table S5*).

As shown in *Table 3*, in the 132 HF-free and stroke-free participants with AF, incident HF and incident stroke were associated with a faster WS decline, as compared with the participants never experiencing such conditions.

**Table 3:** Association between incident HF and incident stroke (as time-varying variables) and WS in AF participants without baseline HF, stroke, and dementia, accounting for the competing risk of death (joint models)

	Incident HF change/year	Incident stroke change/year
	*β* (95% CI)	*β* (95% CI)
Crude	−0.029 (−0.043; −0.015)	−0.031 (−0.046, −0.015)
Model 1	−0.027 (−0.041; −0.014)	−0.030 (−0.045, −0.015)
Model 2	−0.026 (−0.040; −0.013)	−0.030 (−0.047; −0.016)

Model 1: age + education + sex.

Model 2: age + education + sex + hypertension + chronic obstructive pulmonary disease + incident HF + incident stroke + BMI + T2DM + physical activity + alcohol consumption.

AF, atrial fibrillation; BMI, body mass index; CI: confidence interval; HF, heart failure; T2DM, type 2 diabetes mellitus; WS: walking speed.

In terms of anticoagulation treatment, both OAC users and non-OAC users had a steeper decline in WS than participants without AF (*Table 4*). Baseline characteristics of OAC and non-OAC users are shown in Supplementary material online, *Table S6*.

**Table 4:** Association between use of OAC and WS decline accounting for the competing risk of death (joint models)

	Intercept *β* (95% CI)	Annual WS change (95% CI)
Crude model		
No AF (ref.)	–	–
AF no OAC	−0.310 (−0.366; −0.255)	−0.008 (−0.015, −0.002)
AF with OAC	−0.186 (−0.262; −0.110)	−0.014 (−0.023, −0.005)
Model 1		
No AF (ref.)	–	–
AF no OAC	−0.143 (−0.195, −0.091)	−0.008 (−0.015; −0.002)
AF with OAC	−0.030 (−0.110; 0.049)	−0.015 (−0.024, 0.006)
Model 2		
No AF (ref.)	–	–
AF no OAC	−0.040 (−0.090; 0.008)	−0.010 (−0.016; −0.004)
AF with OAC	0.015 (−0.063, 0.094)	−0.014 (−0.022; −0.004)

Model 1: age + education + sex.

Model 2: age + education + sex + hypertension + chronic obstructive pulmonary disease + HF + stroke + dementia + BMI + diabetes + physical activity + alcohol consumption.

AF, atrial fibrillation; BMI, body mass index; CI: confidence interval; HF, heart failure; OAC, oral anticoagulant therapy; WS: walking speed.

## Discussion

Our long-term population-based cohort study showed that AF constitutes an independent risk factor for functional mobility decline over a 15-year follow-up, regardless of age, sex, education, lifestyle factors, BMI, and relevant comorbidities. The detrimental effect of AF on WS decline over time persisted in all the clinical and demographical pre-specified subgroups; nonetheless, it was stronger in participants without a history of stroke at baseline and in people with a higher baseline-reported physical activity. Additionally, over the 15-year follow-up period, among AF participants who were free from HF, dementia, and stroke at baseline, the occurrence of incident stroke and incident HF during the follow-up period was associated with a more rapid decline in motor function. Finally, we observed that the sustained decline in physical function among individuals with AF was not influenced by the use of anticoagulation therapy. These findings underscore the importance of recognizing AF as a significant contributor to the long-term deterioration of physical function, suggesting that interventions targeting AF management and its associated risk factors could potentially mitigate this decline. Our results align with the few previous studies showing a link between AF and motor impairment.^8,14^ However, the extended follow-up period, the utilization of joint models accounting for the competing risk of death, and the focus on incident events provide a more comprehensive perspective on the temporal evolution of this relationship.

Atrial fibrillation and physical frailty intersect through shared pathways, mutually influencing each other. Frailty appears to significantly impact the management and trajectory of AF, while AF may serve as an indicator of frailty.^4,22–24^ Frailty is a syndrome characterized by high biological vulnerability, decreased physiologic reserve, and reduced capacity to resist stressors, due to multiple impairments in inter-related systems, leading to reduced homeostatic reserve. Despite the pressing need for evidence to inform on functional trajectories and prognosis of individuals with AF, tools for a better definition of the pre-frail state and its evaluation are lacking.^17^ Recent investigations have highlighted a growing use of frailty instruments, showing moderate to good inter-rater reliability,^25,26^ but a consensus on widespread implementation is lacking. The concept of frailty holds significant clinical implications. As demonstrated in the study by Diemberger *et al*.,^27^ physicians’ perceptions of frailty in AF patients vary, primarily influenced by age, sex, and weight, and notably differ from the results of objective frailty assessments. In this context, WS is widely recognized as a reliable proxy for physical frailty. Intriguingly, individuals with AF initially exhibiting a WS greater than 0.8 m/s were more prone to decelerate their pace into the lowest group compared with their non-AF counterparts. Furthermore, once participants transitioned to WS levels below 0.8 m/s, their susceptibility to mortality increased (*Figure 1*). This finding was further elucidated through joint modelling, reaffirming the robust association between WS and mortality over time, in line with prior research,^28^ underscoring a diminished risk of death in AF patients with higher walking distance and pace. In our study, individuals without stroke at baseline, showing higher physical activity, were less affected by AF in terms of WS decline. This observation could be attributed to the well-established adverse effects of stroke on physical functionality.^29,30^ On the other hand, a sensitivity analysis carried out in individuals with a WS faster than 0.5 m/s did not confirm the significant effect-modifying role of history of stroke and physical activity on WS, suggesting the crucial impact of baseline functional status on physical mobility decline.

Hence, for patients with AF, regular and comprehensive assessment of cognitive abilities is advisable to prevent functional decline and deterioration in motor performance. Apart from stroke prevention and rhythm management, additional multidisciplinary preventive strategies, such as lifestyle modifications and early detection of these conditions, might alleviate the burden of motor function decline among aging populations with AF. This is aligned with recommendations in current guidelines, for a holistic or integrated care approach to AF management^31^ where adherence to the Atrial fibrillation Better Care (ABC) pathway is associated with improved clinical outcomes.^32^ Ongoing studies, such as the AFFIRMO^33^ and the EHRA-PATH,^34^ which concentrate on outcomes among multimorbid patients receiving polypharmacotherapy, have the potential to offer a thorough assessment for people with AF. Specifically, they can address functional domains and establish a common interdisciplinary approach, fostering collaboration among geriatricians, cardiologists, rehabilitation specialists (e.g. physiotherapists), and primary care physicians to enhance the quality of life for this unique patient population.

In our sample, AF was significantly associated with incident stroke and incident HF; moreover, individuals developing those two conditions demonstrated a more pronounced WS decline compared with the control group, particularly those with stroke, underscoring the potential impact of cerebral disease on physical function.

In light of these results, early identification of high-risk stroke patients is pivotal in promptly initiating motor support activities and more importantly, to assess the correct assumption of OAC.^35^ Indeed, one of the primary hypotheses regarding the physical impairment caused by AF posits that it results from either overt or silent ischaemic strokes, which can damage the cortical and subcortical regions responsible for motor control.^7–9^ Consequently, the utilization of OAC could theoretically reduce the incidence of stroke and thereby decrease the occurrence of declining functional mobility. In the present study, despite of the expected benefit on stroke, functional capacity, cognitive impairment, and dementia,^15,24,28,36^ the use of OAC did not significantly influence the WS decline over time.

This conflicting result can be attributed to several reasons. Firstly, the study population had low rates of baseline anticoagulant prescription (25%), consistent with the previous findings from the Danish National Hospital registry^37^ demonstrating that the proportion of older AF patients prescribed vitamin K antagonists ranged from 13 to 23% between 1995 and 2002. Additionally, the low prescription rate seems to reflect the well-established, common undertreatment with OAC in older individuals with AF.^38^ Furthermore, in older individuals with AF receiving OAC in the early 2000s, treatment quality was frequently suboptimal. This was highlighted in a study by McCormick *et al*.,^39^ which found that only one-fifth of the patients achieved a time in therapeutic range ≥65%, with less than half maintaining an international normalized ratio within the therapeutic range of 2.0 to 3.0. Notably, non-anticoagulated individuals were older with a higher comorbidity burden and had substantially slower WS at baseline relative to anticoagulated counterparts, thereby potentially circumscribing their capacity for further physical performance gains. These results align with a previous study^23^ reporting the negative association between OAC and frailty.

Moreover, these results are impacted by a small sample size, and thus, evaluating the effect of OAC on motor function in larger cohorts or randomized clinical trials would be warranted. Notwithstanding, there are still conflicting data on the effect of OAC in older persons on reducing AF-related incident white matter lesions,^40^ which have been proven to be related to physical function.^41^ Taken together, these findings suggest that while OAC usage is relevant, baseline health status significantly influences WS decline, emphasizing the multifaceted nature of the relationship between AF and physical function.

## Strengths and limitations

To our knowledge, this represents the first cohort study attempting to evaluate the effect of AF on the physical decline throughout a 15-year follow-up period, accounting for the impact of incident diseases and OAC usage in a population-based cohort. This research extends our knowledge of the independent effect of AF on WS decline in older, community-dwelling individuals.

The study has some limitations. First, concerning AF assessment, we could not differentiate between paroxysmal and permanent AF; however, the risk associated with poor outcomes seems to present across various clinical presentations of the arrhythmia. In this regard, data from an observational study by Boriani *et al*.^42^ reported a higher mortality risk in asymptomatic vs. symptomatic patients in terms of risk of stroke, cardiovascular, and all-cause mortality.^25^ Yet, we might not have captured patients with asymptomatic, paroxysmal AF during the follow-up. Furthermore, our study encountered limitations regarding the uncertainty duration of AF, and we recognize that our study may focus on comparing mobility decline between patients with known AF and those without AF. This aspect bears significant importance as the burden of AF has been correlated with diminished quality of life,^43^ heightened risks of cardiovascular hospitalization, ischaemic stroke, and mortality.^30^ However, by evaluating prevalent AF cases, we may have overlooked the emergence of new AF cases, leading to a potential misclassification of incident AF as non-AF. Consequently, this could have diluted the strength of association between the presence of AF and WS. Additionally, the study population from central Stockholm consisted of individuals with a higher socio-economic status than average Sweden, which may not be representative of all older adults. This may limit the generalizability of the findings to the broader population of older adults. Moreover, the study spanned a 15-year period, during which there may have been changes in the management of AF and its associated comorbidities. Specifically, the OAC prescription standards might have been changed during follow-up, also due to the introduction of the direct oral anticoagulants. Similarly, we did not have an adherence measure of the treatment; indeed, more information on OAC use would help us to establish a greater degree of accuracy on the relationship between anticoagulant use and physical performance decline. Additionally, despite extensive adjustments and sensitivity analyses that statistically equalized the two groups, residual confounding may still exist, particularly considering the baseline population differences. For example, we did not include obstructive sleep apnoea, which is associated with both AF and physical function decline, as a covariate in our analysis. Therefore, larger multicentre cohorts of patients with diagnosis of new AF compared with stably confirmed that non-AF individuals are needed to confirm our findings. Finally, the study’s observational nature limits its ability to establish causality. While associations between AF and WS decline, stroke, HF, dementia, and mortality were previously identified, it should be interpreted cautiously as causation cannot be definitively proven.

## Conclusion

Atrial fibrillation is associated with a substantial decline in physical function over time in older adults living in the community. The development of HF and ischaemic events significantly contributes to greater motor function decline in patients with AF, while the use of anticoagulant therapy does not appear to be crucial in preventing this decline in physical performance.

## Supplementary Material

euae173_Supplementary_Data

## Data Availability

Data are available upon reasonable request. Data are from the SNAC-K Project, a population-based study on ageing and dementia (http://www.snac-k.se/). Access to these original data is available to the research community upon approval by the SNAC-K data management and maintenance committee. Applications for accessing these data can be submitted to Maria Wahlberg (Maria.Wahlberg@ki.se) at the Aging Research Center, Karolinska Institutet.
