# Supplementary material for: Atrial fibrillation accelerates functional decline in older adults: a 15-year follow-up population-based study
Source: Europace. 2024 Jun 24;26(7):euae173. doi: 10.1093/europace/euae173 (PMC11273222; doi:10.1093/europace/euae173)
Supplement: euae173_Supplementary_Data [file euae173_supplementary_data.docx]

**Supplemental Figure 1. Study flowchart.**

**
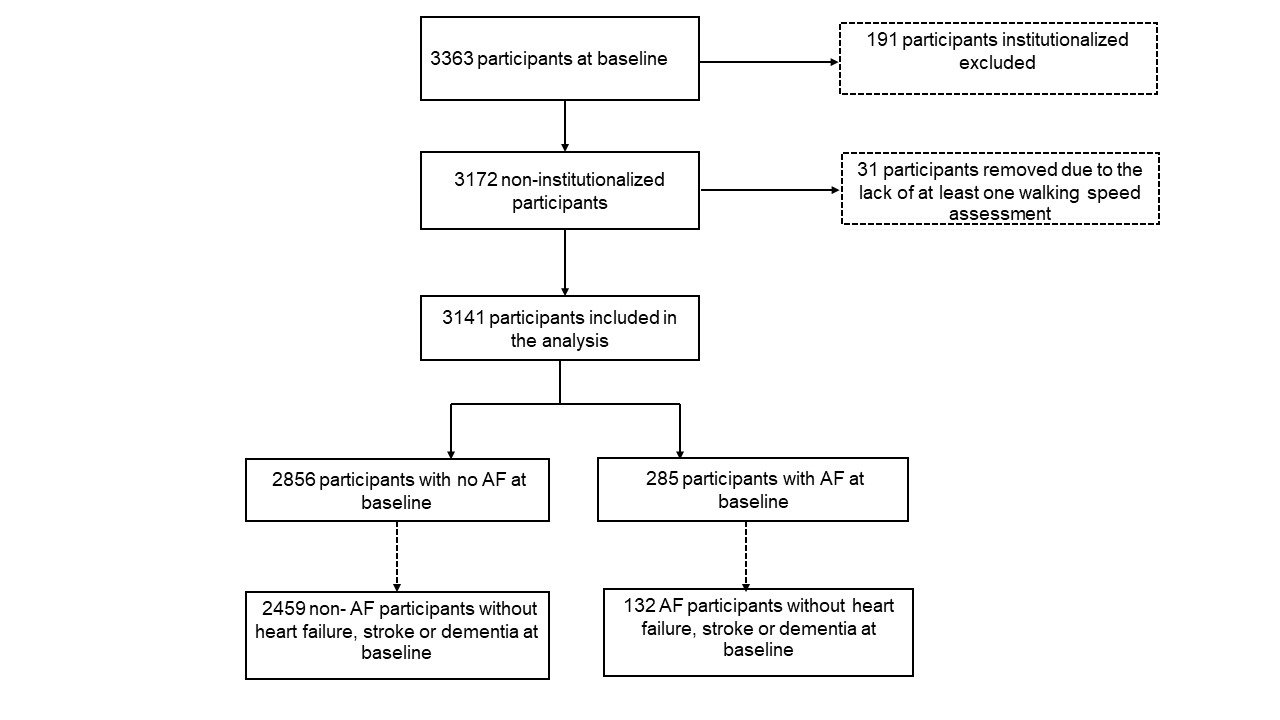
**

**Supplemental Table 1. Sensitivity analysis. Propensity score adjusted joint model between AF and walking speed.**

|  | Intercept  β (95%CI) | Change/year  β (95%CI) |
| --- | --- | --- |
| Crude | -0.32 (-0.27; -0.22) | -0.010 (-0.015, -0.004) |
| PS adjusted | -0.27 (-0.32; -0.22) | -0.013 (-0.018, -0.007) |

*Abbreviations:* PS: Propensity Score adjusted

**Supplemental Table 2. Association between walking speed and mortality from joint models.**

| Models | WS β effect on mortality (95%CI) | Hazard Ratio (95%CI) |
| --- | --- | --- |
| Crude model | -3.20 (-3.39; - 3.00) | 0.040 (0.033; 0.050) |
| Model 1 | -3.53 (-3.73; -3.32) | 0.029 (0.024; 0.036) |
| Model 2 | -3.44 (-3.65; -3.25) | 0.032(0.026; 0.038) |

Model 1: age + education + sex

Model 2: age + education + sex + hypertension + COPD + HF + stroke + dementia + BMI + diabetes + physical activity

*Abbreviations*: BMI: body mass index, COPD: Chronic Obstructive Pulmonary Disease, CKD: Chronic Kidney Disease, HF: Heart Failure, WS: walking speed

**Supplemental Table 3.** Sensitivity analysis. Z- and -p values table for interaction effects between walking speed and history of stroke among participants with walking speed faster than 0.5 m/s

|  | Beta Coefficient | Std. Error | z-value | p-value |
| --- | --- | --- | --- | --- |
| Intercept | 1.1574 | 0.0074 | 156.053 | <0.0001 |
| Time | -0.0340 | 0.0007 | -47.5302 | <0.0001 |
| History of stroke | -0.1863 | 0.0373 | -4.997 | <0.0001 |
| Time x AF | -0.0221 | 0.0030 | -7.395 | <0.0001 |
| Time x AF x History of stroke | -0.0032 | 0.0084 | -0.3787 | 0.7049 |

*Abbreviations:* AF: atrial fibrillation

**Supplemental Table 4.** Sensitivity analysis. Z- and -p values table for interaction effects between walking speed and physical activity among participants with walking speed faster than 0.5 m/s

| Dementia | Beta Coefficient | Std. Error | z-value | p-value |
| --- | --- | --- | --- | --- |
| Intercept | 0.9913 | 0.0158 | 62.881 | <0.0001 |
| time | -0.0342 | 0.0007 | -47.907 | <0.0001 |
| FE-PA | 0.1361 | 0.0177 | 7.6830 | <0.0001 |
| I-PA | -0.2975 | 0.0204 | 14.5570 | <0.0001 |
| Time x AF | -0.0226 | 0.0057 | -3.9600 | 0.0001 |
| Time x AF x FE-PA | -0.0016 | 0.0063 | -0.2479 | 0.8042 |
| Time x AF x I-PA | -0.0032 | 0.0070 | -0.4507 | 0.6522 |

*Abbreviations:* AF: atrial fibrillation; FE-PA: Fitness enhancing physical activity; I-PA: Inadequate physical activity

**Supplemental Table 5.** Association between AF and AF-incident events. Multivariable logistic regression.

|  | Incident dementia | | Incident stroke | | Incident heart failure | |
| --- | --- | --- | --- | --- | --- | --- |
|  | OR (95%CI) | **p-value** | OR (95%CI) | **p-value** | OR (95%CI) | **p-value** |
| Crude | 1.62 (1.11 - 2.25) | **0.0067** | **1.89 (1.28 - 2.26)** | **0.0005** | **2.34 (1.74 - 3.04)** | **<0.0001** |
| Model 1 | 1.16 (0.80 - 1.60) | 0.3780 | 1.63 (1.09 - 2.30) | 0.009 | 1.75 (1.30 - 2.27) | <0.0001 |
| Model 2 | 1.17 (0.80 – 1.60) | 0.3748 | 1.63 (1.09 - 2.311) | 0.009 | 1.76 (1.32 - 2.27) | <0.0001 |

Model 1: age + education + sex

Model 2: age + education + sex + hypertension + COPD + BMI + T2DM + physical activity

*Abbreviations*: AF: atrial fibrillation, BMI: body mass index, COPD: Chronic Obstructive Pulmonary Disease, HF: heart failure, T2DM: type 2 diabetes.

**Supplemental Table 6: Characteristics of study population according to OAC usage and individuals without AF**

|  | AF with OAC (N=71) | AF without OAC (N=213) | NO AF (N=2843) |
| --- | --- | --- | --- |
| **Age, Mean (SD)** | 78.4 (7.79) | 82.2 (9.83) | 73.0 (10.5) |
| **Female sex n(%)** | 33 (46.5%) | 129 (60.6%) | 1827 (64.3%) |
| **Education, n (%)** |  |  |  |
| **Elementary** | 10 (14.1%) | 50 (23.5%) | 452 (15.9%) |
| **High school** | 37 (52.1%) | 117 (54.9%) | 1391 (48.9%) |
| **University** | 23 (32.4%) | 42 (19.7%) | 993 (34.9%) |
| **Physical activity, n (%)** |  |  |  |
| **Inadequate** | 26 (36.6%) | 101 (47.4%) | 827 (29.1%) |
| **Health enhancing** | 36 (50.7%) | 86 (40.4%) | 1391 (48.9%) |
| **Fitness enhancing** | 9 (12.7%) | 26 (12.2%) | 625 (22.0%) |
| **Body Mass Index (BMI), n (%)** |  |  |  |
| **BMI < 18.5** | 2 (2.8%) | 13 (6.1%) | 64 (2.3%) |
| **BMI > 18.5 - 25** | 23 (32.4%) | 90 (42.3%) | 1200 (42.2%) |
| **BMI > 25** | 40 (56.3%) | 85 (39.9%) | 1420 (49.9%) |
|  |  |  |  |
| **# Comorbidities, median [IQR]** | 6.00 [2.5] | 6.00 [3] | 3.00 [3] |
| **Hypertension, n (%)** | 55 (77.5%) | 144 (67.6%) | 1991 (70.0%) |
| **Heart Failure, (n%)** | 35 (49.3%) | 85 (39.9%) | 177 (6.2%) |
| **Stroke, n (%)** | 19 (26.8%) | 36 (16.9%) | 158 (5.6%) |
| **Dementia, n (%)** | 1 (1.4%) | 28 (13.1%) | 125 (4.4%) |
| **COPD, n (%)** | 6 (8.5%) | 18 (8.5%) | 127 (4.5%) |
| **Diabetes mellitus, n(%)** | 14 (19.7%) | 24 (11.3%) | 238 (8.4%) |
| **Chronic kidney disease, n (%)** | 32 (45.1%) | 121 (56.8%) | 901 (31.7%) |
| **Walking speed, mean (SD)** | 0.801 (0.389) | 0.688 (0.446) | 1.02 (0.444) |
| **Death, n (%)** | 8 (11.3%) | 22 (10.3%) | 1358 (47.8%) |

*Abbreviations:* AF: atrial fibrillation*;* OAC: oral anticoagulant therapy
